# Evolving flocking in embodied agents based on local and global application of Reynolds’ rules

**DOI:** 10.1371/journal.pone.0224376

**Published:** 2019-10-29

**Authors:** Rita Parada Ramos, Sancho Moura Oliveira, Susana Margarida Vieira, Anders Lyhne Christensen

**Affiliations:** 1 Instituto Superior Técnico (IST), Lisbon, Portugal; 2 Instituto Universitário de Lisboa (ISCTE-IUL), Lisbon, Portugal; 3 Instituto de Telecomunicações, Lisbon, Portugal; 4 Embodied Systems for Robotics and Learning at the Mærsk Mc-Kinney Møller Institute, University of Southern Denmark (SDU), Odense, Denmark; University of Vermont, UNITED STATES

## Abstract

In large scale systems of embodied agents, such as robot swarms, the ability to flock is essential in many tasks. However, the conditions necessary to artificially evolve self-organised flocking behaviours remain unknown. In this paper, we study and demonstrate how evolutionary techniques can be used to synthesise flocking behaviours, in particular, how fitness functions should be designed to evolve high-performing controllers. We start by considering Reynolds’ seminal work on flocking, the *boids model*, and design three components of a fitness function that are directly based on his three local rules to enforce local separation, cohesion and alignment. Results show that embedding Reynolds’ rules in the fitness function can lead to the successful evolution of flocking behaviours. However, only local, fragmented flocking behaviours tend to evolve when fitness scores are based on the individuals’ conformity to Reynolds’ rules. We therefore modify the components of the fitness function so that they consider the entire group of agents simultaneously, and find that the resulting behaviours lead to global flocking. Furthermore, the results show that alignment need not be explicitly rewarded to successfully evolve flocking. Our study thus represents a significant step towards the use of evolutionary techniques to synthesise collective behaviours for tasks in which embodied agents need to move as a single, cohesive group.

## Introduction

Flocking is a collective behaviour that can be observed in numerous species, such as birds, fish and locusts [1–3]. There are several evolutionary hypotheses for the emergence of flocking behaviour in biological systems. These include safety from predators, which itself can be attributed to different reasons [4]: flocking can confuse predators [5], provide increased vigilance [6, 7], and decrease the individual’s chance of being caught, since it is predominantly the ones at the periphery that are at risk [8]. Another advantage of flocking is foraging efficiency: many studies have shown that individuals find food faster when foraging in a group than on their own [4, 9–11]. Other hypotheses for flocking behaviour in animals include mating success and energy efficiency [12–14].

Flocking became a popular subject of study in domains beyond biology after the seminal work of Reynolds [15], in which he proposed a computational model of flocking called the *boids* model. Reynolds formally defined flocking behaviour as “polarised, non-colliding, aggregate motion”, and showed that three simple rules are sufficient to obtain flocking: (i) cohesion, (ii) separation, (iii) alignment. With these rules, each individual moves towards the centre of mass of its neighbours, while trying to avoid collisions and to align its orientation with the average orientation of its neighbours. Flocking has since become an area of study in several fields, such as statistical physics, control theory, and robotics [16–20].

The ability to flock and display coordinated motion is essential in many tasks for large-scale systems of embodied agents, such as robot swarms. Robot swarms are large groups of autonomous collaborating robots with relatively simple hardware and decentralised control [21]. Inspired by swarm intelligence principles, the main properties of swarm robotics systems are robustness, scalability and flexibility [22]. Control for a swarm robotics system can either be achieved by hand-coded design methods or via automatic design methods. Evolutionary robotics (ER) is a field dedicated to the study and development of methods based on evolutionary computation for automatic synthesis of autonomous robots [23].

The potential of ER to automate the design of behavioral control has been demonstrated in a large number of studies, in which different collective behaviours have been successfully evolved, such as aggregation, dispersion, and foraging [24–28]. However, there are only few studies in evolutionary robotics that have been applied to achieve flocking, and it is still unclear how high-performance flocking behaviours can be reliably synthesised by means of artificial evolution [29].

To date, flocking in artificial embodied agents has primarily been achieved with hand-programmed control [19, 30–32]. The synthesis of such behaviours with ER could pave the way for the evolution of other application-oriented behaviours that rely on flocking. There are many real-world tasks, such as patrolling and environmental monitoring, where groups of autonomous robots sometimes are required to simultaneously exhibit flocking and a task-specific behaviour. In such scenarios, it may be non-trivial to manually design behavioural control that integrates flocking rules with the required task behaviour, and ER can thus be a valuable alternative.

In this paper, we study how evolutionary techniques can be used to synthesise neural controllers for self-organised flocking behaviours. In particular, we adapt the work of Reynolds [15] and translate his three rules to components of the fitness function. Our goal is engineering-oriented, and this study thus represents a step toward the use of ER for collective behaviours in which agents can either benefit from or are required to move as a single, cohesive group.

## Related work

Zaera et al. [29] were among the first to report on attempts to evolve controllers that could exhibit behaviours similar to flocking, schooling or herding, but they did not succeed. Zaera et al. intended to develop neural network-based sensorimotor controllers for schooling behaviour in three dimensions, using realistic Newtonian kinematics, such as inertia and drag. The authors tried to evolve behaviour based on the definitions of flocking suggested in the literature (such as Reynolds’ rules [15]), namely by rewarding individuals for moving at the same speed or for maintaining a certain distance to their neighbours, but they only succeeded in evolving simple group behaviours: aggregation and dispersion.

The authors expressed difficulty in formulating an explicit fitness function that is able to measure the degree of schooling exhibited by a group of agents, claiming that schooling behaviours arises from complex interactions of a number of factors, which are hard to capture in a simple fitness function. The authors furthermore claimed that it can be more difficult to evolve schooling than using manual design methods. It should be noted, however, that Zaera et al. [29] neither specified the fitness functions that were tried, nor did they provide a detailed and quantitative analysis of the obtained behaviours.

Instead of trying to explicitly encode flocking in the fitness function, Ward et al. [33] had more success than Zaera et al. [29], claiming that the obtained results “*would have been difficult*, *if possible at all*, *to foresee or even implement*” with manual design methods. In their work, predators and prey coevolved to produce flocking behaviour. The fitness function is based on the virtual energy levels of each agent, and agents consume or gain energy by performing a given behaviour, such as moving, eating, and mating. At the end of a generation, the individuals with highest energy levels are selected for reproduction. This simple and implicit fitness function led to some interesting results, where preys learned to get close to one another, as well as to the food, and predators learned to pursue preys. However, despite the interesting results, it is not clear to which degree flocking was displayed, since no metrics were applied to quantify the degree of cohesion and alignment among the preys. The authors tried to provide a detailed and quantitative analysis of their work, but they only reported the Euclidean distance between the agents and the food, which gives little information about the degree of cohesion and none about alignment. In fact, the images in the paper show multiple fragmenting groups of preys, instead of a single cohesive flock moving in a common direction.

Baldassarre et al. [27] evolved behavioural control that enabled a swarm of four simulated Khepera robots to aggregate and move as a group toward a light source. Although the study is not strictly focused on flocking, it is actually one of the most frequently cited papers in ER about flocking behaviour, since flocking emerged in one of the experiments. The authors formulated the fitness function with two components, a *compactness* component that promotes cohesion, and a *speed* component that promotes movement in the direction of a light source. From a set of 10 evolutionary runs, a variety of strategies emerged, including flocking in one out of the 10 runs conducted. By analysing the flocking strategy, the authors observed that evolved individuals had different behavioural roles, such as leader and followers. The authors concluded by highlighting the potential of artificial evolution for automating the design of the desired behaviours, which contradict the findings of Zaera [29].

In a more recent contribution, Witkowski and Ikegami [34] evolved neural networks able to display flocking behaviour in a foraging task via signalling. As in Ward et al. [33], the authors used an implicit fitness function that is based on a virtual energy level of each agent. The agents spend energy when moving or emitting signals, and gain energy when eating. The signalling between the agents promoted the flocking behaviour and helped establish temporary leader-follower relations. Similar works can be found in [35–38], in which flocking was not explicitly rewarded in the fitness function, but rather evolved to study a biological hypothesis, mostly involving prey-predators scenarios. Another relevant work that should be highlighted is [39], in which flocking behaviour was demonstrated on physical robots operating under real-world conditions. However, while evolutionary techniques were used, they were only used as a means to optimise specific behavioural parameter values, and not to evolve flocking from scratch.

Overall, there are only few studies that report on attempts at evolving flocking behaviour in evolutionary robotics, and the conditions necessary to evolve flocking behaviour for a swarm of autonomous robots are still unclear. In particular, it remains unexplored how to design fitness functions to explicitly and effectively reward flocking behaviours. In this paper, we show evolutionary techniques can be used to automatically synthesise robot controllers for flocking behaviour, using a simple, explicit fitness function inspired by Reynolds’ rules.

## Materials and methods

In this study, we use a circular differential drive robot model with a diameter of 10 cm. The two wheels enable the robots to move at speeds up to 10 cm/s. Our robot model does not have inertia and a robot can thus rapidly change its speed and orientation. Each robot is equipped with limited onboard sensors: one alignment sensor to perceive the neighbours’ average orientations, and four robot sensors to detect the neighbours’ distance, both with a range of 25 cm.

The alignment sensor computes the orientation of the robot relative to the average orientation of its neighbours within the range of the sensor. The sensor’s value is [0, 1]. If the robot has the same orientation as its neighbours, the reading sensor has a value of 0.5. Otherwise, if the average orientation is in ]0, *π*] or in ]− *π*, 0[, the reading sensor is between [0, 0.5[ and ]0.5, 1], respectively.

The four robot sensors provide information about the distance to the closest neighbour in four sections around the robot. Each sensor has an opening angle of 90° and a range of 25 cm. The reading of a sensor depends linearly on the distance of the closest robot within the sensor’s field of view, according to the following equation:

$$R=1-\frac{d_{n}}{range}$$ (1)

where *d*_*n*_ is the distance to the closest neighbour.

We use an unbounded environment. The robots are initially placed at random initial positions and with random orientations within a square. The side length in metres of the square depends on the number of robots according to the following formula: $\frac{1}{5}\sqrt{\frac{\#\text{robots}}{5}}$, which means that the robots start relatively close to one another, but with no guarantee that every robot is within the sensor range of its nearest neighbour. In our experiments, we used different configurations of swarm sizes, namely 5, 8, 11 and 16 robots, to promote the evolution of general and scalable behaviours.

The experiments were conducted in simulation using JBotEvolver [40], a Java-based open-source, multirobot simulation platform and neuroevolution framework. The use of open-source software allows the experiments in this paper to be replicated and extended by other researchers. The code, the configuration files, and the results can be found at [41].

### Controller architecture and evolutionary algorithm

In evolutionary robotics, artificial neural networks (ANNs) are often used as robotic controllers [42]. As input, the ANN takes normalised readings from the robot’s sensors and the ANN’s output control the robot’s actuators. In our experiments, we use a homogeneous controller corresponding to a continuous time recurrent neural network (CTRNN) [43], with three layers of neurons: a reactive input layer fully connected to a hidden layer, which, in turn, is fully connected to an output layer. The input layer has one neuron for each sensor input and the output layer has one neuron per actuator. Thus, our CTRNN has a total of five input neurons (four robot sensors and one alignment sensor), two output neurons (one for each of the two wheel actuators), and a hidden layer of 10 neurons.

The parameters of the neural network, such as the connection weights, are then optimised by an evolutionary algorithm, to synthesise controllers. We use a simple generational evolutionary algorithm with a population size of 100 genomes. Each genome is evaluated over 24 samples: six samples for each of the four variations of swarm sizes (5, 8, 11 and 16 robots). Each sample lasts 3000 simulation time steps, which is equivalent to 300 seconds. After all the genomes have been evaluated, the individuals’ mean fitness scores are used for selection. The five genomes with highest mean fitness score are selected to become part of the next generation and to populate it with offspring. The genotype of an offspring is the result of applying a Gaussian noise (mean: 0, st. dev: 1) to each gene with a probability of 10%.

The fitness function has to reward the individuals that exhibit flocking. We therefore adopt Reynolds’ seminal work [44], since he successfully defined three simple rules that can achieve flocking. We transform Reynolds’ three rules respectively for *cohesion*, *separation* and *alignment* to components of the fitness function as follows:

$$F=\sum_{t=0}^{time-steps}\frac{\left(C_{t}+S_{t}+A_{t}\right)}{T}+M,$$ (2)

where the first three components correspond to Reynolds’ rules: *C*—cohesion, *S*—separation, *A*—alignment. Although these three components are sufficient to evolve flocking, we considered an additional and optional component for movement, *M*. The components, *C*, *S*, *A*, and *M*, are detailed below.

**Cohesion component (*C*)**: rewards each robot for moving to the centre of mass of their neighbours, as in Reynolds’ rule:

$$C=\frac{1}{N}\sum_{i=1}^{N}(1-\frac{d_{i}}{R})$$ (3)

where *N* is the number of robots, *d*_*i*_ the Euclidean distance a given robot is from its *i*’th neighbour, and *R* the sensor range (25 cm).

**Separation component (*S*)**: penalises robots for colliding with each other, similar to Reynolds’ rule of avoiding collisions with nearby flockmates.

$$S=-\frac{1}{N}\sum_{i=1}^{N}s_{i}$$ (4)

$$s_{i}=\{\begin{array}{cc} 1 & \;\text{if}\;\text{collided}\\ 0 & \;\text{otherwise} \end{array}$$ (5)

where *N* is the number of robots.

**Alignment component (*A*)**: rewards each robot for matching its orientation to the average of their neighbours, as Reynolds suggests “*steer towards the average heading of local flockmates*” [44]:

$$A=\frac{1}{N}\sum_{k=1}^{N}\psi_{k}$$ (6)

$$\psi_{k}=\frac{1}{F}∥\sum_{a=1}^{F}e^{i\theta_{a}}∥$$ (7)

where *N* is the number of robots, *F* the number of flockmates of a individual *k* (including itself), ∥…∥ denotes the norm of a vector and *θ*_*a*_ is the orientation of the corresponding flockmate *a*. This component first computes the average orientation of each robot with its nearby neighbours. Each robot obtains a reward between [0, 1], having a value of 1 when the neighbourhood has a common direction, and a value of 0 when there is no alignment (or no neighbours). Then, *A* is computed as the mean score of all individual rewards.

The above components only reward the swarm for cohesion, separation and alignment. Thus, when conducting our initial set of experiments, flocking did emerge, but around the robots’ initial positions (see S1 Video), since the fitness function does not reward robots for moving farther away. Therefore, we consider an additional and optional component for movement, *M*, that promotes the swarm to move away from the initial positions:

$$M=\{\begin{array}{cc} d/D & \;\text{d}\;\leq \;\text{D}\\ 1 & \;\text{otherwise} \end{array}$$ (8)

where *D* is a distance that the swarm should move from the initial location (5 m) and *d* the actual distance the robots moved, on average. When *d* > *D*, *M* is set to a value of 1, thereby *M* is defined in [0, 1]. Rather than just rewarding the robots for moving farther away, this term could as well be adapted to enable flocking for a specific task, such as area coverage or foraging.

In our experiments, the fitness scores are calculated offline: after all the data of the swarm is available. Note, however, that it could be easily adapted to be implemented as an online approach, namely by having each robot compute its fitness and then aggregate their results to obtain the final fitness score.

### Metrics

Flocking behaviour is usually defined as an aggregate and polarised motion [15], where individuals move together in a common direction. Thus, in order to evaluate the evolved behaviours, we used metrics that measure the alignment and cohesion of the swarm, similar to other works in robotics [19, 20].

**Order**: To evaluate the degree of alignment among the individuals, we used the order metric, well known both in robotics and control theory [19, 20, 45]:

$$\psi =\frac{1}{N}\cdot ∥\sum_{k=1}^{N}e^{i\theta_{k}}∥$$ (9)

where *θ*_*k*_ is the orientation of individual *k*. This metric averages the orientation of the *N* robots, having a value of 1 when the swarm is aligned, and value of 0 when there is no common direction.

**Number of Groups**: As to evaluate flock cohesion, we adapted the metric *number of group* suggested by Ferrante in [20]. Ferrante defined this metric as the number of groups at the end of the experiment. To define what constitutes a group, Ferrante used equivalences classes {*x* ∈ *N* ∣ *x* ∼ *a*}, with *N* being the set of all robots, *a* an element of the set, and ∼ the neighbourhood equivalence relation. More concretely, the distances between all pairs of robots are computed, and then, with a given distance threshold (in our work: 25 cm which corresponds to the range of the sensors), the subsets of robots that share the same neighbourhood are found, making each different subset a new equivalence class. For instance, robot *A* and *B* are neighbours if their distance is smaller than the defined threshold, and if *B* is also neighbour of *C*, this results in an equivalence class constituted by robots *A*, *B* and *C*. In the end, the total number of of groups is equal to the number of equivalence classes.

**Swarm Cohesion**: Evaluating flock cohesion just in terms of group splits can be limiting, given that it is also important to consider the number of robots in each subgroup. When the swarm splits, it is preferable that only a few robots leave the main group, compared to the main group spliting into two or more large subgroups. Considering this, we used an additional metric that computes the proportion of robots that remain in the main group:

$$1-\frac{N_{rl}}{N}$$ (10)

where *N*_*rl*_ is the number of robots that leave the main group, i.e. the group with the majority of the robots.

## Results and discussion

In each experimental setup, a total of 30 evolutionary runs were conducted, each lasting 6000 generations. For the highest-scoring controller evolved in each run, we conducted a post-evaluation with 400 samples, 100 for each of the four combinations of number of robots (5, 8, 11 and 16 robots). In this section, we present the results obtained, and we analyse the performance and behaviours of the evolved solutions according to the metrics presented in Section Metrics.

### Experimental setup inspired by Reynolds’ local rules—*Local setup*

**Fig 1:**
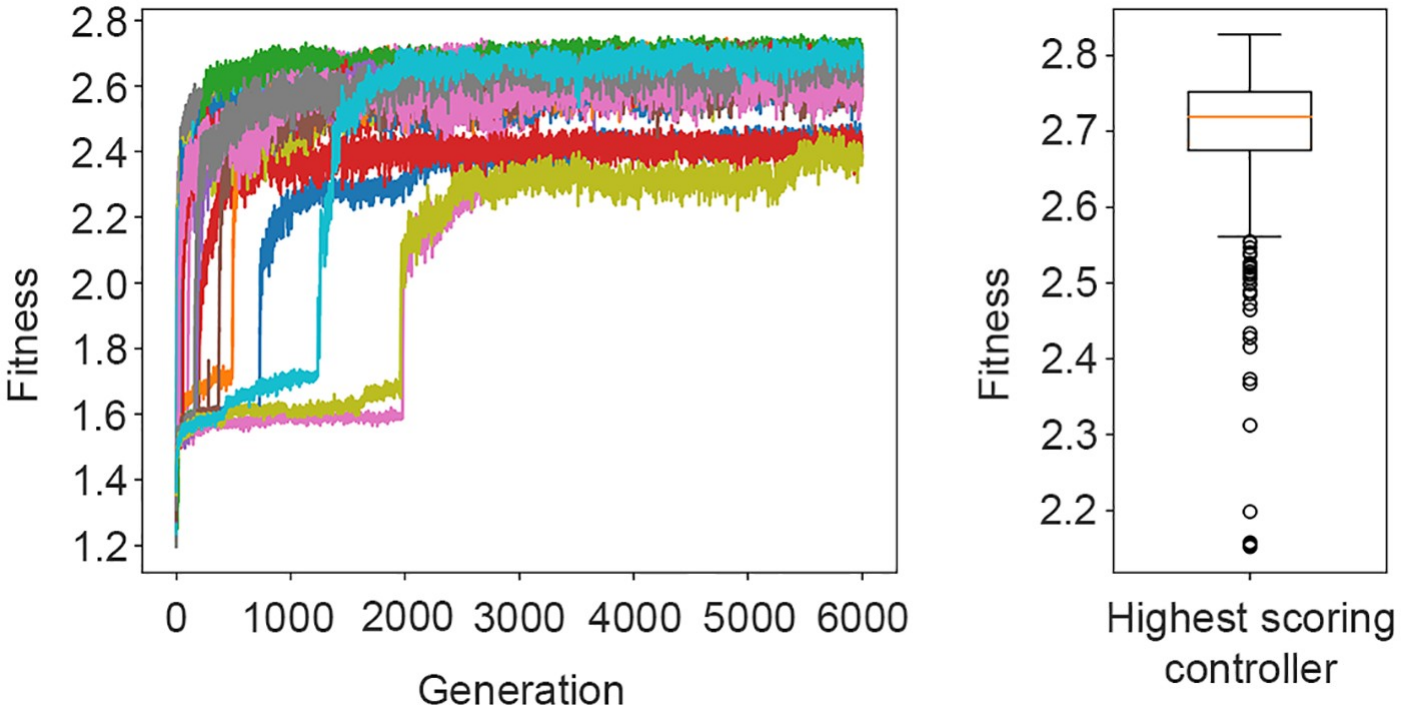
Left: Fitness trajectories of the highest-scoring controllers evolved in each evolutionary run. Right: Distribution of the post-evaluation fitness scores achieved by the highest-scoring controller of the 30 evolutionary runs conducted. The box-plot summarises results from 400 post-evaluation samples.

Fig 1(left) shows the fitness trajectories of the 30 evolutionary runs. The highest-scoring controller obtained an average fitness of 2.70 ± 0.10 during post evaluation, see Fig 1(right).

**Fig 2:**
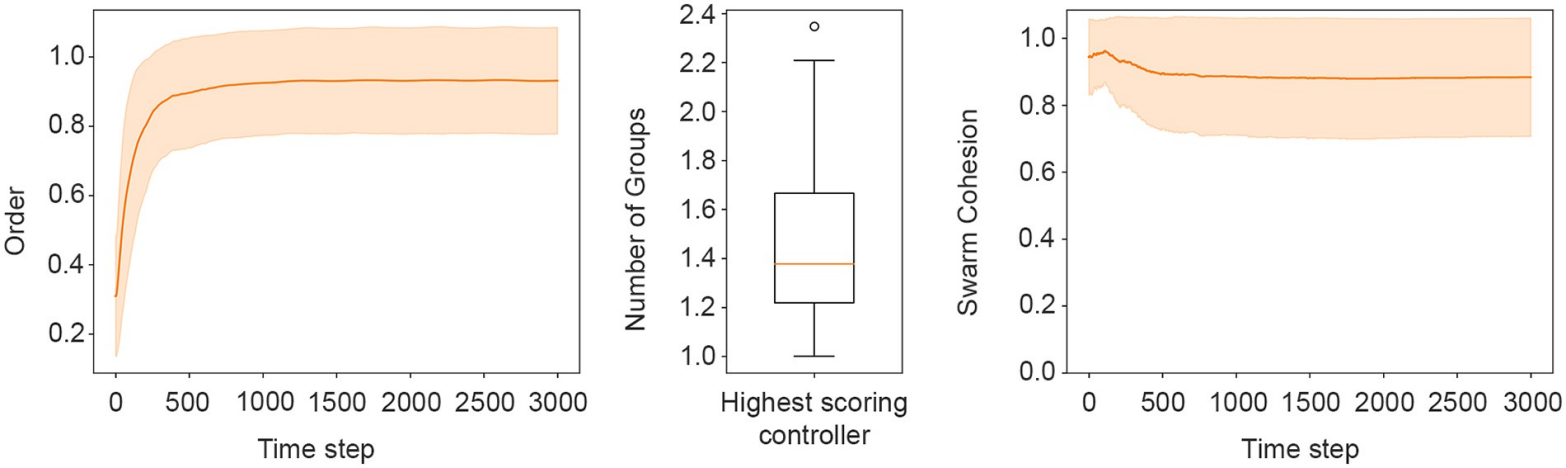
The figure summarises results of the average from the 400 post-evaluation samples of the highest-scoring controller in terms of the metrics presented in Section Metrics. Left: The *order* metric trajectory across the 3000 simulation steps. Centre: box-plot of the distribution of the *number of groups* metric. Right: The *swarm cohesion* trajectory across the 3000 simulation steps. The shaded areas indicate the standard deviation.

Fig 2(left) summarises the performance of the highest-scoring controller in terms of alignment (the *order metric*). The results show that the evolved solution scored a mean value of 0.90 ± 0.09 during the experiment. Fig 2(centre) shows the performance of the highest-scoring controller with respect to the *number of groups* metric. The evolved solution had a mean *number of groups* of 1.42 ± 0.31 at the end of the experiment, meaning that the swarm splits into two groups in almost half of the samples. Fig 2(right) summarises the performance of the highest-scoring controller in terms of the *swarm cohesion* metric. The highest-scoring controller achieved a mean value of 0.89 ± 0.02.

**Fig 3:**
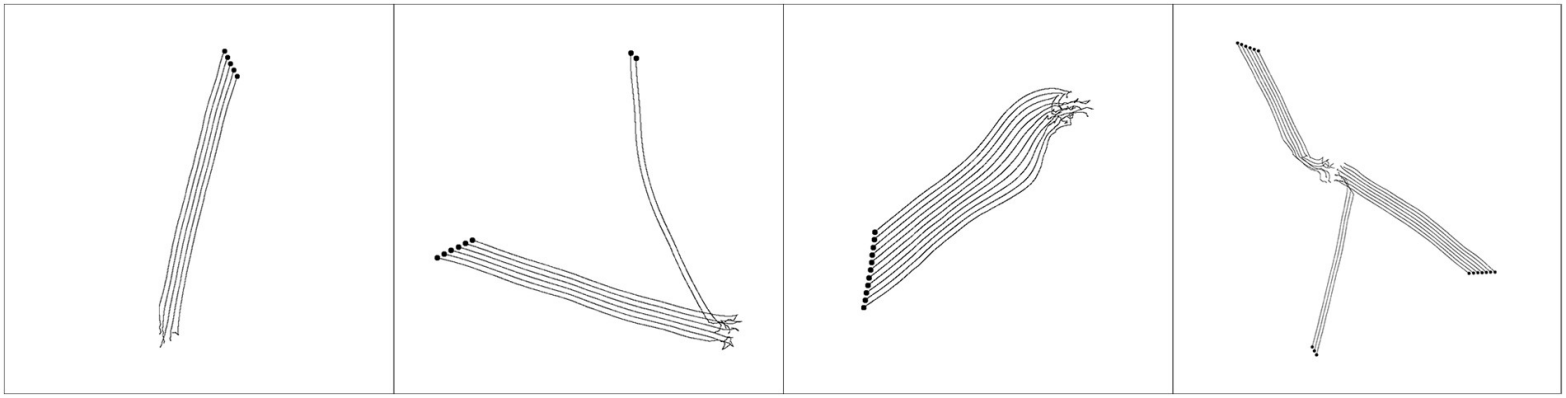
Example of the behaviour displayed by the highest-performing controller, with 5, 8, 11 and 16 robots, from left to right. The lines represent the trajectory of the robots.

Examples of evolved behaviours for the four different configurations of number of robots (5, 8, 11 and 16) can be seen in Fig 3. The examples show how the evolved behaviour often leads to the emergence of local flocks, as the quantitative results also revealed. These results can be explained by the fact that the fitness function only promotes local cohesion and local alignment, since it was adapted from the local rules of Reynolds: robots learn to flock, but only with their immediate neighbours, and not with the entire swarm.

We also experimented with different weights on the alignment component and on the cohesion component. In a series of independent experiments, we multiplied weights of respectively 0.0, 0.25, 0.5, and 0.75 on one of the two components, while the other component had no weight multiplied, and vice versa. In all the evolved behaviours, we observed the same result, namely that the swarm quickly fragments into smaller, local flocks.

Since the fitness function inspired by Reynolds’ local rules only scores behaviour with respect to immediate neighbours, we decided to conduct an additional set of experiments in which the fitness function takes into account the global behaviour (the whole swarm), rather than only local interactions (neighbours).

### Experimental setup scoring global behaviour—*Global setup*

In this section, we reformulate the fitness function so that it scores global behaviour, a technique that is widely applied in evolutionary robotics to favour the evolution of cooperative behaviours [46, 47]. The new fitness function, *F*^*g*^, is based on global alignment and global cohesion, defined as follows:

$$F^{g}=\sum_{t=0}^{time-steps}\frac{\left(C_{t}^{g}+S_{t}+A_{t}^{g}\right)}{T}+M,$$ (11)

**Alignment component (*A*^*g*^)**: scores the orientation of the swarm, promoting a common direction between all individuals. This term is computed using the same formula of the *order* metric:

$$A^{g}=\frac{1}{N}\cdot ∥\sum_{k=1}^{N}e^{i\theta_{k}}∥$$ (12)

**Cohesion component (*C*^*g*^)**: rewards the swarm for keeping together as a single group:

$$C^{g}=\frac{1}{N\;umbero\;fGroups}$$ (13)

where the term *NumberofGroups* is the same as in Section Metrics. As before, the cohesion component is defined in [0, 1].

Note, that we did not reformulate the separation component since a global version of it would be mathematically equivalent to the local version: the fitness simply corresponds to the total number of collisions divided by the number of robots.

For the new global experimental setup, we used the same configuration of runs, generations and post-evaluation as in the previous setup. When referring to the two setups, we will hereinafter use *Local setup* to denote the setup in which the fitness function, *F*, is based on local application of Reynolds’ rules (the previous section); and *Global setup* to denote the setup in which the fitness function, *F*^*g*^, is based on global application of Reynolds’ rules (this section).

**Fig 4:**
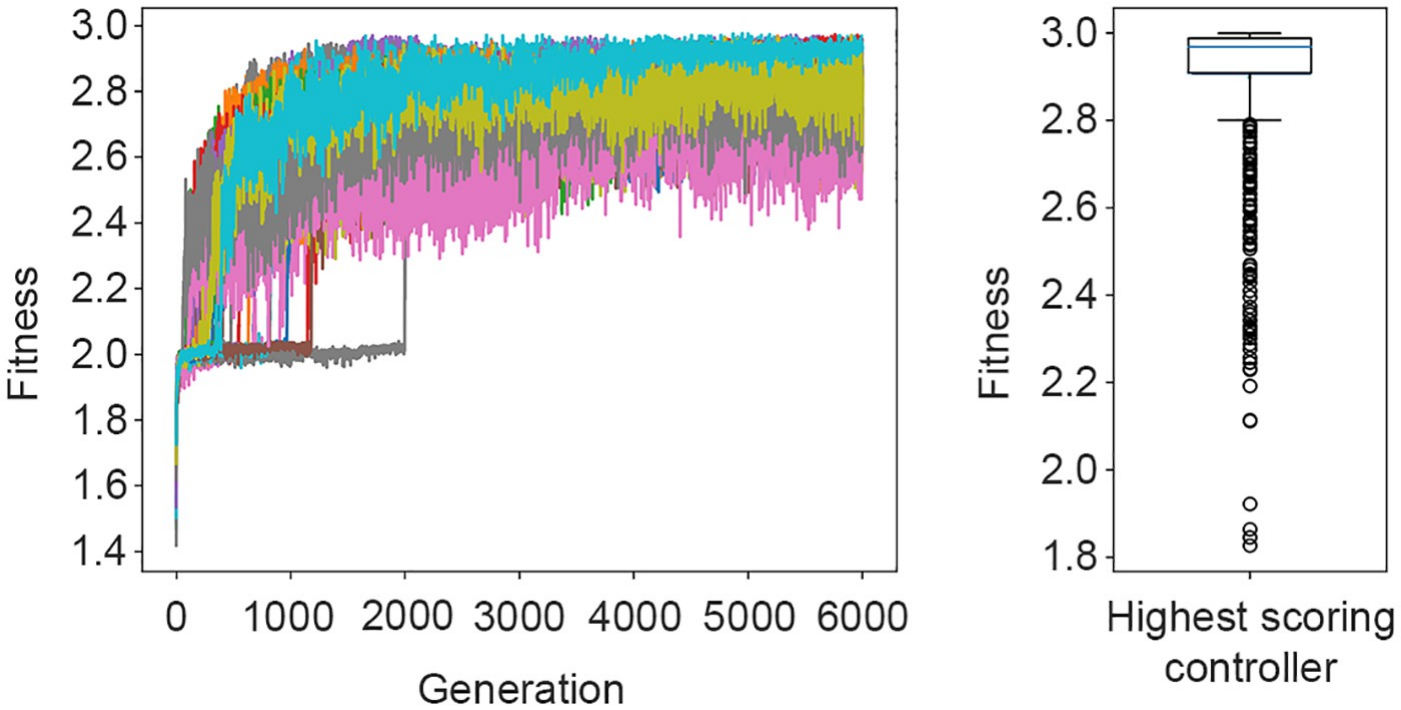
Left: Fitness trajectories of the controllers in each of the 6000 generations. Right: Distribution of the post-evaluation fitness scores achieved by the highest-scoring controller.

The 30 fitness trajectories of the controllers during the 6000 generation can be seen in Fig 4(left). The highest scoring controller achieved an average fitness of 2.87 ± 0.22 in post-evaluation, as shown in the box-plot in Fig 4(right).

**Fig 5:**
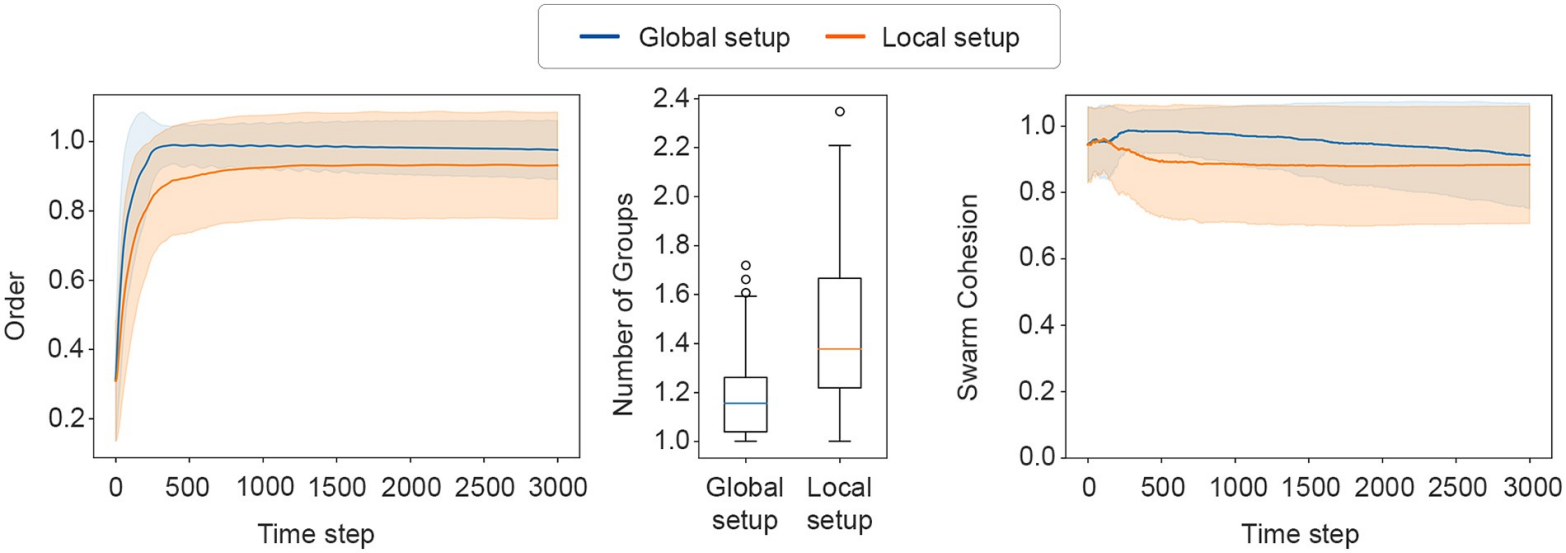
The metric scores achieved by the highest-scoring controllers of *Global setup* and the *Local setup*. Left: the *order* metric. Centre: the *number of groups* metric. Right: the *swarm cohesion* metric. The shaded areas indicate the standard deviation.

Evaluating the performance of the evolved solution, we can observe that the highest-scoring controller outperformed the best one evolved in the *Local setup*, with respect to all metrics, see Fig 5. Regarding alignment, the results show that the evolved solution achieved aligned motion, scoring a mean value of 0.97 ± 0.07, outperforming the 0.90 ± 0.09 of the *Local setup*.

In terms of group splitting, the swarm remains cohesive during the experiments, obtaining a mean score of 1.19 ± 0.18 number of groups. In the *Local setup*, the swarm split into two in approximately half of the samples, whereas in the *Global setup*, by scoring global cohesion, the swarm is generally able to remain a single cohesive group.

**Fig 6:**
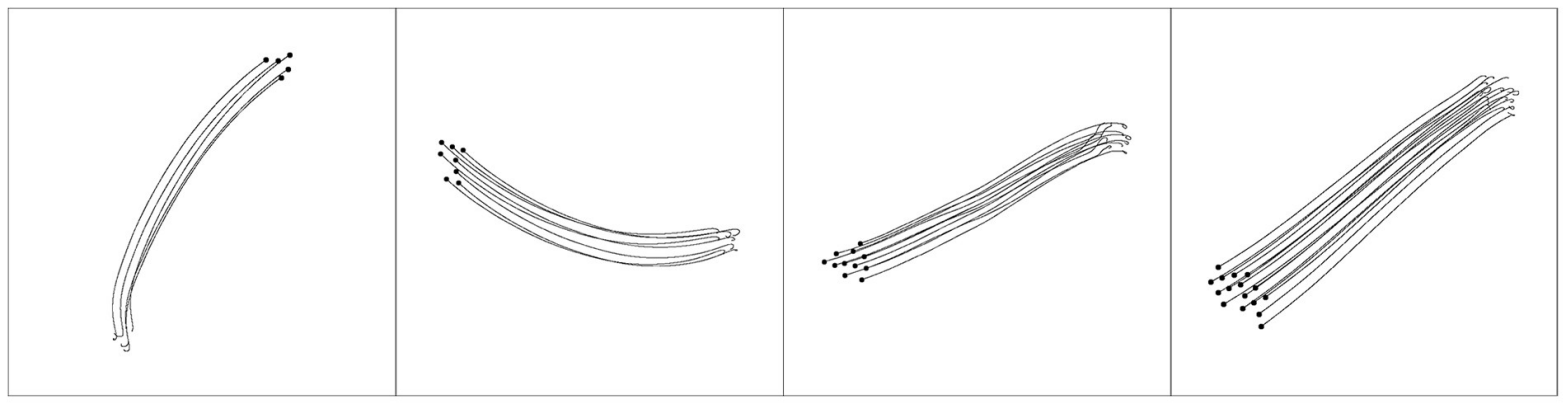
Example of the behaviour displayed by the highest-performing controller evolved in the *Global setup*, with 5, 8, 11 and 16 robots, from left to right. Observing the beginning of trajectory, we can see the robots evolved the strategy of making a quick turn around their initial positions, in order to first form an aligned group before moving away.

As for the *swarm cohesion* metric, the highest-scoring controller achieved a mean value of 0.95 ± 0.02 during the experiment. Observing the plot in Fig 5(right) with respect to the *Global setup*, we can also see an interesting steep increase in the first 300 steps (30 seconds), suggesting that the group size increased. This can be explained by the fact that, in the beginning of the experiment, robots are placed close to each other, but at random positions, which does not ensure that every robot is seen by its nearest neighbours. To cope with this, the robots evolved the strategy of moving in small circles around their initial positions to find their neighbours, and they only then move away from the origin as a single flock (see Fig 6 and S2 Video). This can also be seen in the plot of the *order* metric, for which the swarm also takes 300 steps to achieve alignment, the time needed to ensure that the flock contains all robots and that all robots are aligned before moving away from their initial positions.

**Fig 7:**
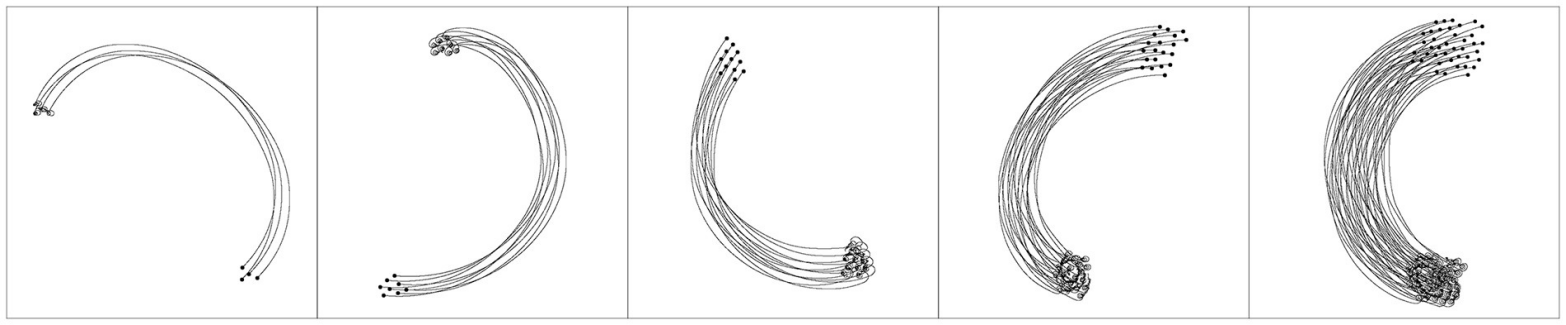
Example of the behaviour displayed in the scalability experiments, with 4, 8, 12, 20 and 40 robots, from left to right.

We conducted an additional set of experiments with larger group sizes, namely with 4, 8, 12, 20 and 40 robots. In particular, these new evolutionary experiments were performed with 6000 generations, each evaluated over 25 samples, i.e. five samples for each of the five swarm sizes, and each sample lasting twice as many steps, 6000 steps (600 seconds), to compensate for the larger swarm size. Given the increased computational complexity of the larger swarm size, we were only able to conduct three evolutionary runs. In the resulting behaviour, we also observe that robots initially coordinate by moving around their initial location in small circles. As can be seen on Fig 7 and S3 Video, robots spend the first part of the sample coordinating their direction of motion before moving away from their initial positions. These are only preliminary experiments, and further work is therefore necessary to study the evolution of flocking behaviour for large numbers of agents, and to analyse the properties of the evolved behaviour in large swarms. Studies involving up to 1000 hand-coded flocking agents have been conducted [19, 30], and it would be interesting to analyse if artificially evolved flocks display behavioural correlations similar to large animal flocks [48, 49].

The results show that the evolved solutions successfully achieved flocking. By adapting the Reynolds-inspired fitness components from local to global behaviour, the resulting solutions display swarm-wide, synchronised and coordinated motion.

After finding that adapting the local rules of Reynolds as global components of a fitness function can successfully lead to flocking, we then studied if the global versions of all of Reynolds’ principles are necessary to embed in the fitness function to evolve flocking. We therefore conducted additional experiments, from which we found that the alignment component is not required to evolve flocking, as we show in the next section.

### Experimental setup without the alignment component—*No Alignment setup*

In this setup, the *No Alignment setup*, the fitness function was formulated to no longer contain the alignment component, hence the fitness function only rewards the robots for moving as a cohesive group.

$$F^{na}=\sum_{t=0}^{time-steps}\frac{\left(C_{t}^{g}+S_{t}\right)}{T}+M,$$ (14)

**Fig 8:**
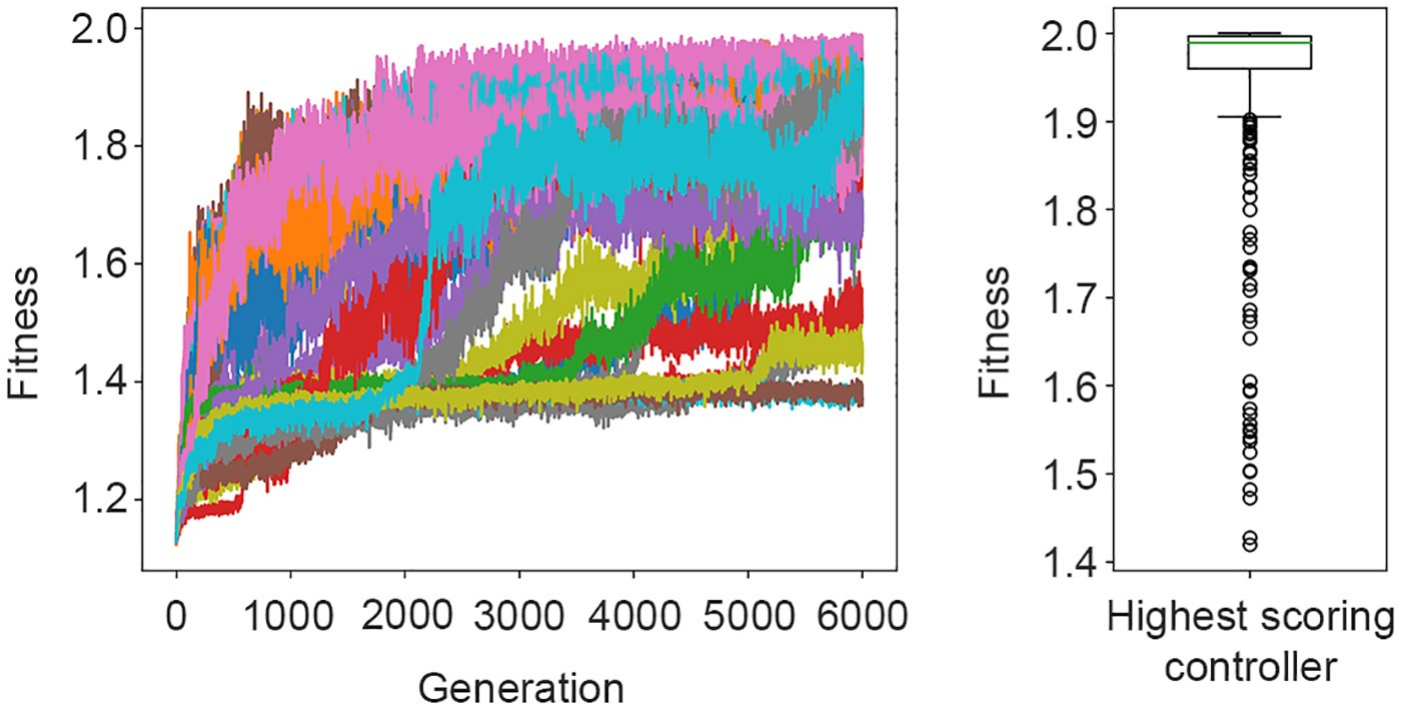
Left: Fitness trajectories of the 30 controllers within 6000 generations. Right: Box-plot of the results of the 400 post-evaluation samples achieved by the highest-scoring controller of the *No Alignment setup*.

The fitness trajectories of the *No Alignment setup* can be seen in Fig 8(left), with the same number of runs and generations as used in the previous two setups. Fig 8(right) summarises the post-evaluation fitness scores of the highest-scoring controller, achieving a mean fitness of 1.95 ± 0.11 (note that the fitness no longer rewards alignment, and therefore has a theoretical maximum value of 2 instead of 3 as in the previous setups).

**Fig 9:**
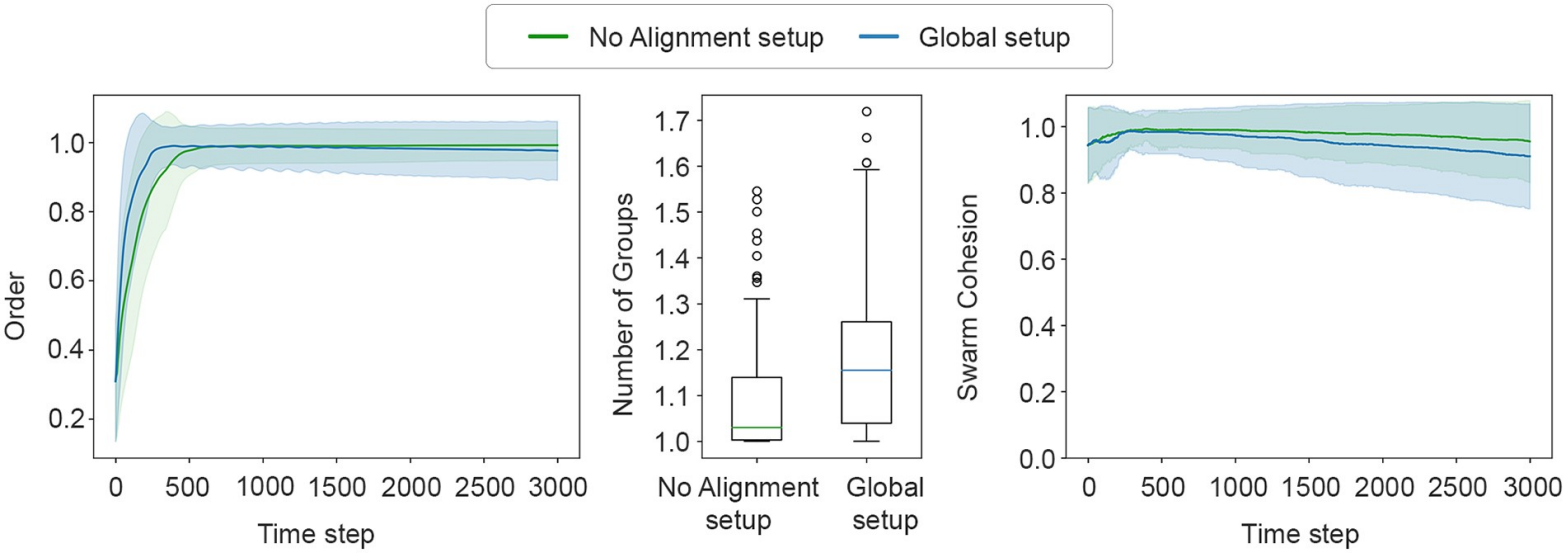
The metric scores achieved by the highest-scoring controller of the *No Alignment setup* and the *Global setup*. Left: The *order* metric. Centre: the *number of groups* metric. Right: the *swarm cohesion* metric. The shaded areas indicate the standard deviation.

Even without the alignment being explicitly rewarded in the fitness function, the evolved solution successfully achieve flocking, as can be observed in Fig 9, which shows a similar performance to the previous setup, both in terms of cohesion and alignment.

**Fig 10:**
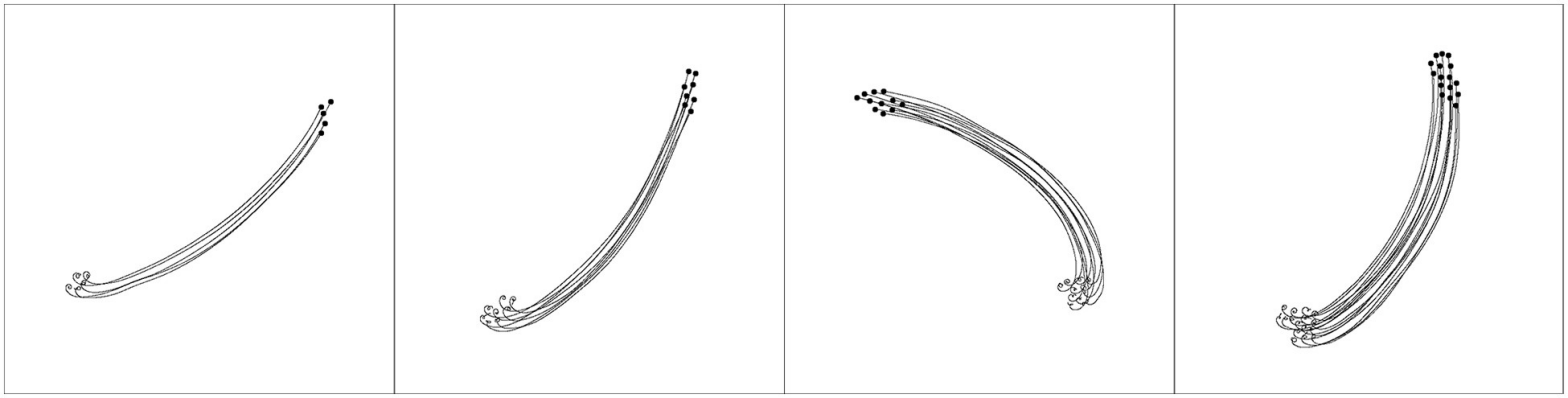
Example of the behaviour displayed by the highest-performing controller of the *No Alignment setup*, with 5, 8, 11 and 16 robots, from left to right. The behaviour displayed is more cohesive than the one in Fig 6.

With respect to alignment, the highest-scoring controller had a mean value of 0.96 ± 0.10, similar to the best evolved in the *Global setup* with 0.97 ± 0.07. As for the *number of groups* metric, the highest-scoring controller obtained a mean value of 1.10 ± 0.14, compared to 1.19 ± 0.18 in the *Global setup*. With respect to the *swarm cohesion* metric, the evolved solution scored a mean value of 0.98 ± 0.01 during the experiment and 0.96 ± 0.12 in the final simulation step, compared to the solution evolved in the *Global setup* that reached 0.95 ± 0.02 and 0.91 ± 0.16, respectively. An example of the behaviour displayed by the highest-performing controller can be seen in Fig 10 and S4 Video. In the beginning of the sample, the robots circle around their initial positions, similar to the solutions evolved in *Global setup*, to ensure cohesion prior to moving. In addition, the flocking behaviour is more compact than in the *Global setup*, which can be explained by the fact the solutions now maximise cohesion, since the fitness function only scores cohesive motion.

Results show that the principle of alignment need not be explicitly rewarded to evolve flocking. Even without scoring alignment, the swarm achieves aligned motion, with each robot changing its orientation in synchrony with its neighbours. This can be explained by the fact that, by just rewarding the swarm for moving as a cohesive group, alignment is promoted, since collective motion is most easily achieved when motion is aligned. Indeed, when a robot changes its orientation at the same time as its neighbours, it remains in range of the rest of the group, resulting in a cohesive swarm.

Similar to evolving flocking without explicit scoring alignment, there are also studies of hand-coded design that did not used explicitly the alignment rule. For a review of the combinations of flocking rules and their impact, see, for instance, [20, 50].

## Conclusions

Inspired by Reynolds’ flocking rules, we explored simple, explicit fitness functions to evolve controllers that effectively demonstrate flocking behaviour. We conducted evolutionary runs in three different setups: (i) We started by adapting Reynolds’ three local behavioural rules to components of the fitness function, which scored separation, cohesion and alignment with respect to each robot’s neighbours within range. Scoring local behaviour resulted in fragmented local flocking. (ii) In our second set of experiments, we adapted the fitness function so that the three components considered the whole swarm, rather than only local interactions. By scoring global behaviour, the evolved solutions successfully displayed self-organised flocking. Our results showed that flocking can be evolved by embedding Reynolds’ local rules in a fitness function, with the condition that the rules are applied globally. (iii) We then studied if components based on all of Reynolds’ rules are necessary to achieve flocking. In the final setup, the fitness function did not explicitly reward alignment, but flocking still emerged. We thus found that flocking can be achieve without explicitly rewarding alignment.

In this paper, we have shown that controllers can be evolved to display flocking, a complex behaviour, with a simple explicit fitness function, contrary to what was claimed in previous work [29]. It would also be interesting to study other explicit fitness functions based on metrics that are commonly used in flocking studies.

In this study, the robots were not subject to inertial effects beyond those stemming from limitations on wheel speed, and they were thus able to rapidly change their speed and orientation. In previous studies, it has been shown that inertia, both on velocity [51] and on behaviour [52] can have a significant and positive impact on flocking performance. In ongoing work, we are studying the evolution of flocking for specific hardware platforms that are subject to significant inertia effects, such as autonomous surface vehicles [26]. We are furthermore studying how to integrate flocking in the evolution of other application-oriented behaviours, such as patrolling and environmental monitoring, that represent real-world tasks that can be undertaken by swarms of autonomous robots.

## Supporting information

S1 Video Example of a behaviour evolved without the movement component. (MP4)

S2 Video Example of an evolved behaviour in the *Global Setup*. (MP4)

S3 Video Example of an evolved behaviour in the scalability experiments. (MP4)

S4 Video Example of an evolved behaviour in the *No Alignment Setup*. (MP4)
